# Supplementary material for: Pyruvate Dehydrogenase Kinase 1 inhibition mediated oxidative phosphorylation enhancement in cartilage promotes osteoarthritis progression
Source: BMC Musculoskelet Disord. 2023 Jul 20;24:597. doi: 10.1186/s12891-023-06585-6 (PMC10357736; doi:10.1186/s12891-023-06585-6)
Supplement: Supplementary file 1 — Additional file 1. [file 12891_2023_6585_MOESM1_ESM.zip › MF.Gsea.1653627316242/GOMF_HISTONE_LYSINE_N_METHYLTRANSFERASE_ACTIVITY.html]

Details for gene set GOMF\_HISTONE\_LYSINE\_N\_METHYLTRANSFERASE\_ACTIVITY[GSEA]

|  || Dataset | 55457\_collapsed\_to\_symbols.cls.cls #OA\_versus\_normal.cls.cls #OA\_versus\_normal\_repos |
| Phenotype | cls.cls#OA\_versus\_normal\_repos |
| Upregulated in class | normal |
| GeneSet | GOMF\_HISTONE\_LYSINE\_N\_METHYLTRANSFERASE\_ACTIVITY |
| Enrichment Score (ES) | -0.43033034 |
| Normalized Enrichment Score (NES) | -1.5052049 |
| Nominal p-value | 0.03322259 |
| FDR q-value | 0.17701282 |
| FWER p-Value | 1.0 |
Table: GSEA Results Summary

  

Fig 1: Enrichment plot: GOMF\_HISTONE\_LYSINE\_N\_METHYLTRANSFERASE\_ACTIVITY      
 Profile of the Running ES Score & Positions of GeneSet Members on the Rank Ordered List

  

| SYMBOL | TITLE | RANK IN GENE LIST | RANK METRIC SCORE | RUNNING ES | CORE ENRICHMENT || 1 | N6AMT1 | N-6 adenine-specific DNA methyltransferase 1 [Source:HGNC Symbol;Acc:HGNC:16021] | 330 | 0.734 | 0.0386 | No |
| 2 | ASH2L | "ASH2 like, histone lysine methyltransferase complex subunit [Source:HGNC Symbol;Acc:HGNC:744]" | 452 | 0.681 | 0.0889 | No |
| 3 | SMYD2 | SET and MYND domain containing 2 [Source:HGNC Symbol;Acc:HGNC:20982] | 856 | 0.553 | 0.1060 | No |
| 4 | SMYD3 | SET and MYND domain containing 3 [Source:HGNC Symbol;Acc:HGNC:15513] | 2361 | 0.319 | 0.0170 | No |
| 5 | SETMAR | SET domain and mariner transposase fusion gene [Source:HGNC Symbol;Acc:HGNC:10762] | 3216 | 0.229 | -0.0293 | No |
| 6 | EZH1 | enhancer of zeste 1 polycomb repressive complex 2 subunit [Source:HGNC Symbol;Acc:HGNC:3526] | 3565 | 0.197 | -0.0391 | No |
| 7 | SETD3 | "SET domain containing 3, actin histidine methyltransferase [Source:HGNC Symbol;Acc:HGNC:20493]" | 4506 | 0.113 | -0.1023 | No |
| 8 | SMYD5 | SMYD family member 5 [Source:HGNC Symbol;Acc:HGNC:16258] | 4769 | 0.089 | -0.1149 | No |
| 9 | SETDB1 | SET domain bifurcated histone lysine methyltransferase 1 [Source:HGNC Symbol;Acc:HGNC:10761] | 4891 | 0.080 | -0.1173 | No |
| 10 | NSD1 | nuclear receptor binding SET domain protein 1 [Source:HGNC Symbol;Acc:HGNC:14234] | 5178 | 0.058 | -0.1344 | No |
| 11 | EHMT2 | euchromatic histone lysine methyltransferase 2 [Source:HGNC Symbol;Acc:HGNC:14129] | 5507 | 0.032 | -0.1571 | No |
| 12 | MECOM | MDS1 and EVI1 complex locus [Source:HGNC Symbol;Acc:HGNC:3498] | 5622 | 0.022 | -0.1641 | No |
| 13 | SUV39H1 | suppressor of variegation 3-9 homolog 1 [Source:HGNC Symbol;Acc:HGNC:11479] | 6028 | -0.007 | -0.1950 | No |
| 14 | KMT5B | lysine methyltransferase 5B [Source:HGNC Symbol;Acc:HGNC:24283] | 6198 | -0.020 | -0.2064 | No |
| 15 | NSD3 | nuclear receptor binding SET domain protein 3 [Source:HGNC Symbol;Acc:HGNC:12767] | 6220 | -0.022 | -0.2060 | No |
| 16 | EZH2 | enhancer of zeste 2 polycomb repressive complex 2 subunit [Source:HGNC Symbol;Acc:HGNC:3527] | 6562 | -0.047 | -0.2285 | No |
| 17 | SETD2 | "SET domain containing 2, histone lysine methyltransferase [Source:HGNC Symbol;Acc:HGNC:18420]" | 6926 | -0.074 | -0.2502 | No |
| 18 | NSD2 | nuclear receptor binding SET domain protein 2 [Source:HGNC Symbol;Acc:HGNC:12766] | 7622 | -0.129 | -0.2929 | No |
| 19 | SUV39H2 | suppressor of variegation 3-9 homolog 2 [Source:HGNC Symbol;Acc:HGNC:17287] | 8529 | -0.196 | -0.3462 | No |
| 20 | KMT2A | lysine methyltransferase 2A [Source:HGNC Symbol;Acc:HGNC:7132] | 8807 | -0.217 | -0.3487 | No |
| 21 | DOT1L | DOT1 like histone lysine methyltransferase [Source:HGNC Symbol;Acc:HGNC:24948] | 8832 | -0.220 | -0.3313 | No |
| 22 | SETD4 | SET domain containing 4 [Source:HGNC Symbol;Acc:HGNC:1258] | 9452 | -0.269 | -0.3558 | No |
| 23 | PRDM16 | PR/SET domain 16 [Source:HGNC Symbol;Acc:HGNC:14000] | 10211 | -0.342 | -0.3848 | No |
| 24 | PRDM9 | PR/SET domain 9 [Source:HGNC Symbol;Acc:HGNC:13994] | 10798 | -0.405 | -0.3948 | Yes |
| 25 | CXXC1 | CXXC finger protein 1 [Source:HGNC Symbol;Acc:HGNC:24343] | 10853 | -0.411 | -0.3630 | Yes |
| 26 | WDR82 | WD repeat domain 82 [Source:HGNC Symbol;Acc:HGNC:28826] | 10854 | -0.411 | -0.3271 | Yes |
| 27 | TTLL12 | tubulin tyrosine ligase like 12 [Source:HGNC Symbol;Acc:HGNC:28974] | 10861 | -0.411 | -0.2915 | Yes |
| 28 | SETD1B | "SET domain containing 1B, histone lysine methyltransferase [Source:HGNC Symbol;Acc:HGNC:29187]" | 11235 | -0.461 | -0.2801 | Yes |
| 29 | KMT2D | lysine methyltransferase 2D [Source:HGNC Symbol;Acc:HGNC:7133] | 11590 | -0.514 | -0.2626 | Yes |
| 30 | EHMT1 | euchromatic histone lysine methyltransferase 1 [Source:HGNC Symbol;Acc:HGNC:24650] | 11686 | -0.530 | -0.2236 | Yes |
| 31 | RBBP5 | "RB binding protein 5, histone lysine methyltransferase complex subunit [Source:HGNC Symbol;Acc:HGNC:9888]" | 11816 | -0.553 | -0.1852 | Yes |
| 32 | SETD5 | SET domain containing 5 [Source:HGNC Symbol;Acc:HGNC:25566] | 12339 | -0.688 | -0.1655 | Yes |
| 33 | ASH1L | ASH1 like histone lysine methyltransferase [Source:HGNC Symbol;Acc:HGNC:19088] | 12405 | -0.708 | -0.1085 | Yes |
| 34 | KMT2B | lysine methyltransferase 2B [Source:HGNC Symbol;Acc:HGNC:15840] | 12446 | -0.723 | -0.0483 | Yes |
| 35 | SETD1A | "SET domain containing 1A, histone lysine methyltransferase [Source:HGNC Symbol;Acc:HGNC:29010]" | 12793 | -0.954 | 0.0083 | Yes |
Table: GSEA details [plain text format]

  

Fig 2: GOMF\_HISTONE\_LYSINE\_N\_METHYLTRANSFERASE\_ACTIVITY      
 Blue-Pink O' Gram in the Space of the Analyzed GeneSet

  

Fig 3: GOMF\_HISTONE\_LYSINE\_N\_METHYLTRANSFERASE\_ACTIVITY: Random ES distribution      
 Gene set null distribution of ES for **GOMF\_HISTONE\_LYSINE\_N\_METHYLTRANSFERASE\_ACTIVITY**
